# Enhancing pedagogical practices with Artificial Neural Networks in the age of AI to engage the next generation in Biomathematics

**DOI:** 10.1007/s11538-025-01511-4

**Published:** 2025-08-31

**Authors:** Jeremis Morales-Morales, Alonso Ogueda-Oliva, Carmen Caiseda, Padmanabhan Seshaiyer

**Affiliations:** 1 Department of Mathematics and Applied Sciences, Inter American University of PR-San German, Puerto Rico, USA; 2 Department of Mathematical Sciences, George Mason University, Fairfax, Virginia, USA; 3 Department of Natural Sciences and Mathematics, Inter American University of PR-Bayamon, Puerto Rico, USA

**Keywords:** C-MATH, biomathematics modeling, MS-Excel, Artificial Neural Networks, 97D20, 97M60, 97P50, 97P80

## Abstract

In this work we present a C-MATH-NN framework that extends a C-MATH framework that was developed in recent years to include prediction using artificial neural networks (NN) in a way that is engaging, interdisciplinary and collaborative to help equip our next generation of students with advanced technological and critical thinking skills motivated by social good. Specifically, the C-MATH framework has successfully helped students understand a real-world *Context* through a mathematical *Model* which is then *Analyzed* mathematically and *Tested* through appropriate numerical methods with data, and finally this undergraduate research becomes a *Habit* for students. Furthermore, the explanation of the main components of a simple NN-model serves as an introduction to this popular artificial intelligence tool. This framework has contributed to the success of talented students in mathematical biology research and their academic goals. We present a visual introduction to the architecture of artificial neural networks and its application to disease dynamics for all interested learners. We introduce a simple feed forward physics-informed neural network (PINN) built in MS-Excel that works very well for an epidemiological model and an equivalent Python implementation that is robust and scalable. The products introduced in this work are shared in an online repository with curriculum material for students and instructors that includes MS-Excel workbooks and Python files to facilitate the acquisition of technology tools to explore and use in their own projects.

## Introduction

Rapid advancement of AI and data analytics is transforming educational landscapes across disciplines, particularly in mathematical biology (Bodine et al. 2014; Chiel et al. 2010; Farrior et al. 2007; Kot 2003; Reed 2004; Robeva and Laubenbacher 2009). This intersection of biology, mathematics, problem solving, and data science presents unique opportunities for educators to improve pedagogical practices, promoting engagement and deeper understanding among students (Seshaiyer and Lenhart 2020). From population dynamics to the spread of diseases, the biomathematics community has used mathematical modeling to study increasingly complex biological systems and engage students. When combined with efficient computational tools, mathematical biology offers a fertile ground for impactful projects that cultivate the critical thinking and communication skills required of our workforce in the artificial intelligence (AI) era (NRC 2003; Gravemeijer et al. 2017; Ellerton 2013; Duncan et al. 2014; Dawes et al. 2019). Computational design and systems thinking, collaboration, and communication skills are consistently mentioned in pedagogic circles and industry (Anhalt and Cortez 2014; Campbell 2007; MathWorks 1990; Stein et al. 2008; Sorgo 2010; Suh and Seshaiyer 2015, 2016; Rossi et al. 2004) and fundamental growing our next-generation of scientists.

With the proliferation of data in the biological sciences - thanks to genomic sequencing, ecological monitoring, and healthcare analytics - students can engage with real-world data, enhancing their learning experiences. AI-driven simulations have the potential to replicate complex biological processes, enabling students to experiment with variables in a controlled environment. For example, students can simulate the impact of environmental changes or human behavior on population dynamics, gaining insights into ecological balance or spread of an infection, respectively. By incorporating real datasets, educators can guide students in data analysis and interpretation, fostering critical thinking. Students can analyze epidemiological data to model disease spread, developing a deeper understanding of both mathematical concepts and public health implications.

The integration of data and AI tool into mathematics education cultivates critical thinking through projects/problem based learning. Students question assumptions, validate models against real-world scenarios, and derive meaningful conclusions from their analyses. These critical skills are essential to navigate complex biological issues, such as COVID-19 (Ohajunwa et al. 2020) and complex social challenges such as the opioid epidemic (Singh et al. 2024) and domestic violence (Ohajunwa et al. 2021). The mathematical modeling community has used differential equations to model an ample range of real-world phenomena. This has inspired creative educators to teach differential equations (DE) courses using modeling to engage students, as evidenced by productive organizations such as SIMIODE Winkel (2015), CODEE Greer (2020), and NIMBioS McMichael et al. (2015) to name a few. The authors are a cross- disciplinary community of students and faculty that collaborate in using various modeling projects such as the spread of diseases moderated by human behavior as pedagogic tools (Ogueda-Oliva and Seshaiyer 2024), (Seshaiyer and Caiseda 2023). This community of practice learns from each other’s strengths, communicating interdisciplinary concepts motivated by a common goal: solve complex problems for the good of society. We developed a framework to be used as a Project-Based Learning strategy in a cross-disciplinary setting that provides ample opportunities for students to learn and develop their thinking skills. This innovative educational framework takes advantage of the accelerated use of technology driven by the pandemic to enhance learning in mathematical biology.

In this work, we introduce C-MATH-NN as a composition of the C-MATH pedagogical framework in (Seshaiyer and Caiseda 2023) combined with AI technology of Disease/Physics-Informed Neural Networks (DINN/PINN) using Literate Programming instruction (Shaier et al. 2022; Ogueda-Oliva and Seshaiyer 2024; Raissi et al. 2019a, b). Literate Programming instruction for learning mathematical concepts with technology can be found visiting the GitHub website^1^. C-MATH is an undergraduate research pedagogical framework that produced multiple papers in STEM with students as co-authors. The C-MATH-NN has produced engaging projects in mathematical biology that are rich in the use of data, technologies and teamwork communication. We showcase the development of one of these projects to illustrate the process and its transformational power in the experience of a talented high school student working with a research team. This collaborative and cross-disciplinary arena is also of great value for the educational enrichment of higher level students and their teachers, as shown in references (NRC 2003; Seshaiyer 2012, 2017; Seshaiyer and Kappmeyer 2016).

This work is organized into 5 sections including the introduction in Section 1. In Section 2 discusses the C-MATH pedagogical framework followed in Section 3 by an introduction to neural networks for students, its different architectures and application to disease dynamics. The implementation and performance of the shared source files and code in MS-Excel and Python are included in Section 4, and results and impact of this curriculum are discussed in Section 5.

## Preparing Future Generations to Address Global Pandemics

**Fig. 1:**
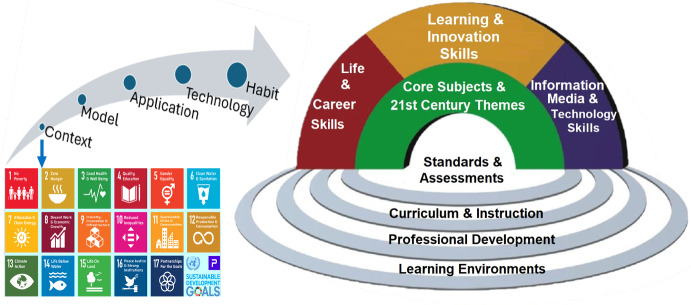
From Context to Habits C-MATH

As mathematicians tackle increasingly intricate real-world issues through research, the imperative of addressing sustainability challenges identified in the United Nations’ Sustainable Development Goals (UN-SDGs) is ever more pressing especially in light of the COVID-19 pandemic *context*. Figure 1 shows several of UN-SDGs that concern the COVID-19 pandemic, including (i) Goal 3: Good Health and Well-being, (ii) Goal 8: Decent Work and Economic Growth, and (iii) Goal 17: Partnership for the Goals. It is important to use the opportunity to train the next generation workforce to address and solve these global challenges through innovative mathematical thinking (Seshaiyer 2025). Introducing students to straightforward yet powerful mathematical *models* offers them a clear entry point into intricate contexts such as COVID-19 and equips them to tackle similar challenging tasks. Sustained learning enthusiasm involves introducing students to do research through *analysis* that nurtures their sense of empowerment. Moreover, students grasp the significance of data by *testing* interpretation, visualization, analysis, and prediction, learning to communicate with user-friendly visual representations—such as dashboards—to comprehend the spread of diseases. Consequently, they draw connections between mathematical research and its potential to tackle challenges outlined in the UN-SDGs which becomes a *habit*. In table 1 COVID-19 is used to illustrate the described C-MATH framework.

**Table 1:** C-Math-NN Framework applied to COVID-19 Model

**C-MATH**	**The COVID-19 Pandemic Problem**
C –Context	United Nations Sustainable Goals interaction: SDG-3 Good health and well-being, SDG-8 Decent Work and economic Growth, SDG-17 Partnerships and empathy among government, industry, health, education stakeholders including human behavior isolation and compliance issues
M -Model	Creative modified SIR models to characterize the COVID-19 pandemic and human behavior. Innovative use of NN for disease modeling
A -Analysis	Mathematics of $\mathcal {R}_0$ proofs, Statistics and NN performance and parameter estimation.
T -Test	Educational impact of teaching early technology and math modeling integrating Data to problem solving. Test math models with simulation, NN parameter estimation, and sensitivity of $\mathcal {R}_0$.
H -Habits	Practicing interdisciplinary research collaboration among social sciences and biomathematics (Project INSIGHT, RAISE: NSF-IHBEM Abstract No. 2230117). In Education: student oriented research, communication, teamwork, critical thinking on strengths and weaknesses of a model, technical mindset to organize and divide problems into ordered components, mathematical concept definitions and proofs, and DS skill for exploration, analysis and discovery

### C-MATH mathematical biology Research for Student Learning

The importance of research projects to engage students and facilitate learning cannot be overstated. In "Expanding Undergraduate Research in Mathematics: Making UR more inclusive", the Mathematical Association of America published work (Seshaiyer and Caiseda 2023), we shared multiple examples of undergraduate research projects that have transformed students of all backgrounds, including minorities, and even their mentors. Global challenges based on the UN-SDGs create empathy that transcends local and cultural barriers providing interdisciplinary and cross-cultural perspectives that enrich thinking. From Context to Habit, the C-MATH framework, summarizes those educational ideals that are pursued in the development of the required mathematical biology skills in a global society for our next generation in the following way.

Start with *Context, Context, Context*! The UN-SDG is a collection of 17 international goals that are interconnected and aim to serve as a blueprint for a better and sustainable future for people, prosperity and our planet. For example, Good Health and Well-being goal (SDG-3) is associated to the pandemic and interconnected with Decent Work and Economic Growth (SDG-8) and Partnerships for the Goals (SDG-17) as our world raced to find solutions and shared data to help curb the spread of the disease, and later deal with the consequences of severe government restrictions that have impacted economy, education and well-being.

Developing a *Model* is an opportunity to formulate and discover concrete relationships through data analysis and mathematical tools that are fit to characterize complex phenomena in a simplified form. The model is also a growing mathematical object that can have multiple evolving versions in the modeling activity as the researchers continue to discover. Data-driven models can be attractive for students who formulate pertinent mathematical questions that lead to different levels of understanding of the underlying method as their intellectual curiosity is awakened. Our team was able to evolve the enhanced SEIR COVID-19 model into new ones such as: Explicit SEAIQRD, Implicit human behavior SEAIQRD Model (Ohajunwa et al. 2020) and later by introducing the confinement compartment with domestic violence variables, a compound social problem is modeled (Ohajunwa et al. 2021).

*Analysis* provides the opportunity to introduce mathematical skills and rigor. Different models provide different testing strategies that are shared in literature as best-practices. For example, in the compartmental SIR it is common to study equilibrium, existence, uniqueness, boundedness of the solution, stability, derive the basic reproduction number $\mathcal {R}_0$, final size, and sensitivity of the model.

*Testing* a model is an opportunity to add numerical methods, technology implementations and Data Science skills. With technology tools the model is transformed into visualizations, numerical results, simulations, and the data processing cycle and model improvements are applied.

*Habits* developed through research of real world problems in mathematical biology contexts generate an extraordinary thinking tank as shown in (Simpson 1949). It is an opportunity for cross-disciplinary teams to collaborate, communicate, share ideas, compare and find a common ground. It also opens the discussion to ethical issues that may arise particularly in the use of technology, AI and data management. The intentional use of skills such as empathy, emotional intelligence, design, critical and systems thinking, are invaluable not only for our workforce but also for the individual’s life. The C-Math framework is illustrated in Figure 1.

In a data-informed world accelerated by the AI revolution, students should be educated about the use of popular machine learning data analysis techniques. We have witnessed that artificial neural networks have proven to be very successful and is a good computational tool to teach in a way that we continue to build both the scientific framework and the technological skills.

## Teaching Mathematical Biology with Neural Networks

Since the advent of advanced machine learning techniques, such as artificial neural networks and deep learning and combined with greater computational power the scientific modeling community has speculated that algorithms may be able to learn autonomously anything, provided the required data. However this is not true yet. In 2017, Raissi, Perdikaris and Karniadakis introduced physics-informed neural networks(PINN), neural networks that are trained to solve supervised learning tasks while respecting any given law of physics described by general nonlinear partial differential equations (PDE). PINNs are used nowadays to solve partial differential equations (PDE), fractional equations, and integral-differential equations. (Cuomo et al. 2022; Raissi et al. 2019a). PINNs approximate PDE solutions by training a neural network to minimize a loss function that takes into account the underlying physics including disease transmission equations. (Raissi et al. 2019b).

**Fig. 2:**
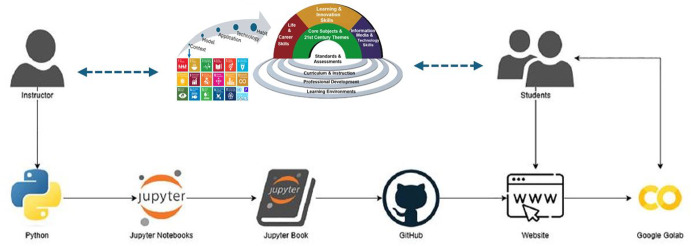
C-MATH with Literate Programming to motivate teaching differential equations with NN-based approaches

Our team has expanded the concept and successfully implemented a disease informed neural network that corresponds to the implicit COVID-19 Model developed using the C-MATH framework (Shaier et al. 2022). The technological tools that lead to the development of a mathematical modeling teaching website using Python and GitHub tools are illustrated in Figure 2. Note that instructors that prefer R can also use Jupyter notebooks, or may choose to substitute Rmarkdown/Bookdown for Jupyter Notebook/Book. The instructor builds an interactive book that is accessed in a website by students so that they can experiment in real time with short scripts of code using Google Colab. The interactive book is built using mathematical learning modules with embedded Python code in a Jupyter notebook. The modules are bind in a Jupyter Book to facilitate Literate Programming instruction, and stored in the instructor’s GitHub. This book is then published on a website where students can access the final product avoiding installation and website navigation hassle.

In the following subsection, we discuss the pedagogy that guides C-MATH-NN.

### An Integrated Instructional Framework for Biomathematics

As the landscape of education rapidly evolves, the integration of technology into teaching demands more than technical know-how. It requires a thoughtful integration of what to teach (Content), how to teach it (Pedagogy), and the tools used to deliver it (Technology). This intersection is captured in the TPACK framework—Technological Pedagogical Content Knowledge—first introduced by Mishra and Koehler (2006). TPACK emphasizes that effective technology integration in education involves not just adding digital tools to traditional instruction, but understanding how these tools reshape content delivery and student learning experiences.

Against this backdrop, C-MATH-NN emerges as a compelling example of a TPACK-aligned pedagogical innovation tailored for the age of AI. It exemplifies the integrated thinking that TPACK encourages as follows:

- T (Technology): C-MATH-NN embraces accessible digital tools through low-code programming in Excel and a popular Python implementation. These platforms enable students to engage with Artificial Neural Networks (ANNs) without requiring extensive programming expertise.
- P (Pedagogy): The model leverages Literate Programming, a pedagogical style where code and explanations coexist within a single, interactive document. This design supports exploratory learning and lowers the entry barrier by eliminating the need for multiple software installations or technical setups.
- C (Content): C-MATH-NN is inherently interdisciplinary, weaving together mathematical modeling, biomedical topics (such as epidemiological modeling), and AI concepts. It introduces learners to core principles of neural networks while grounding them in real-world, socially relevant applications.
- K (Knowledge): The knowledge gained through the C-MATH framework equips learners with both conceptual understanding and workforce-relevant skills. It empowers them to interpret and simulate AI models using practical, industry-relevant tools, addressing the growing demand for AI literacy across disciplines.

By uniting T, P, and C knowledge in a balanced and meaningful way, C-MATH-NN not only reflects the TPACK framework but extends its relevance into future-ready education. It serves as a model of how educators can leverage technology not as an add-on, but as an integrated, transformative force in teaching and learning in the age of AI.

To introduce students to PINNs, we first engage them in a “low” code environment through a "Simple-PINN" using spreadsheets such as MS-Excel that work well before introducing a “high” code implementation with more robust Python tools that introduce interested students to these technologies that continue to become more popular with the aid of AI-assistants to flatten the learning curve.

### An Introduction to Artificial Neural Network

**Fig. 3:**
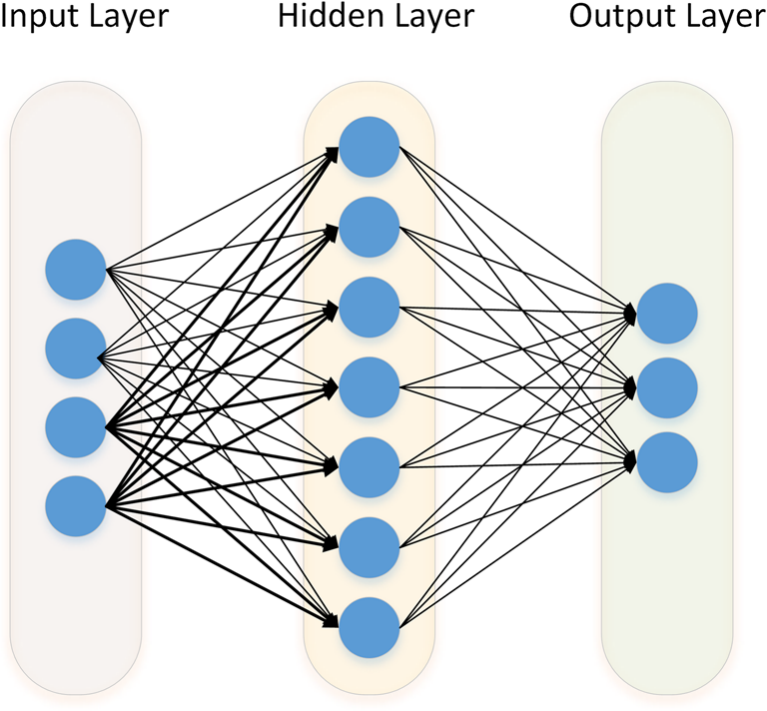
Feed Forward Neural Network visualization

Artificial neural networks are a class of machine learning models for classification, regression predictions, pattern recognition, and more. They consist of interconnected nodes organized into layers. The input layer receives raw data, while the output layer produces the final predictions or outputs. In between, there can be one or more layers, known as hidden layers, which extract and transform features from the input data. To obtain the hidden-layer parameters, an optimizer is used to minimize the error between the real and predicted data. Nodes in the hidden layer or *neurons* receive the input signals, compute an operation, and produce an output signal that may pass to nodes in the next layer. This is known as a feedforward NN, the simplest non-recurrent structure available illustrated in Figure 3.

Neurons are the basic building blocks of neural networks. Each neuron applies a transformation to the input data using an activation function to produce an output. To explore the inner workings of a NN, let us consider a basic neural network with a ReLu activation function that is a nonlinear function that outputs the maximum value between 0 and the input.

**Fig. 4:**
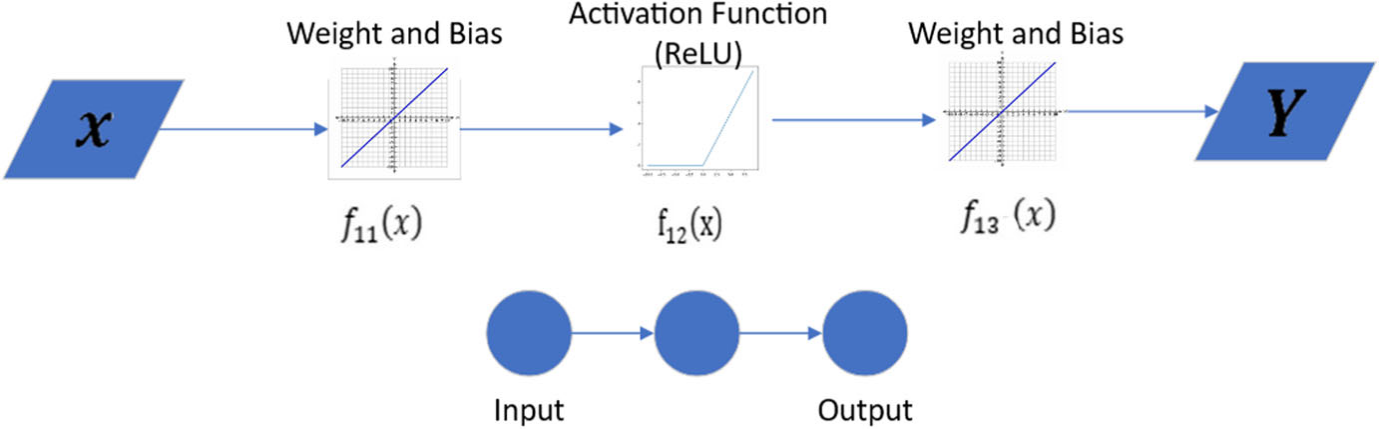
Feedforward NN with one input node, one neuron with ReLU activation function and one output node. Here *x* is the input vector, $f_{ij}(x)=w_{ij}x+b_{ij}$. Here $i= 1$ is the neuron number, $j \in$ {1,2,3} is the step, and ReLU activation function $= max(0,f_{ij}(x))$

Let us define $f_{NN} = Y$ in (1) as the neural network composite function that contains weights ($w_i$), biases ($b_i$) and activation function parameters in Figure 4. This illustrates a single neuron NN using a ReLU activation function.

1 $$\begin{aligned} f_{NN} (x)=Y=f_1 \circ f_2 \circ f_3=f_3 (f_2 (f_1 (x)))=w_2 (max(0, w_1 x+b_1 ) )+b_2 \end{aligned}$$

An activation function in the context of neural networks is a mathematical operation applied to the input of a node to produce its output. This function determines whether a neuron should be activated by calculating the weighted sum of its inputs and applying a transformation to it. Activation functions are crucial for introducing non-linear properties into the network, enabling it to learn and model complex data patterns. Functions such as Sigmoid, Tanh, ReLu, and others can be used as activation functions. For other activation functions and more complex NN see the supplementary material in Ogueda-Oliva et al. (2025).

**Fig. 5:**
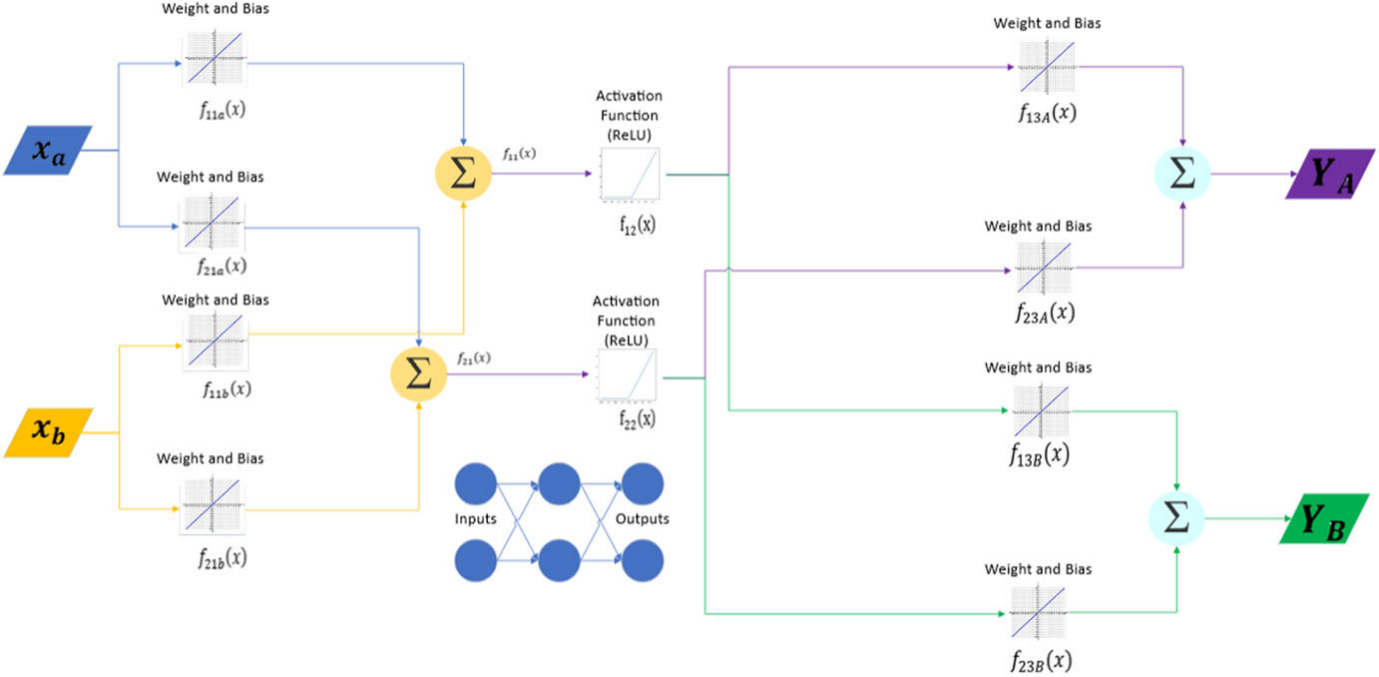
NN with two inputs nodes, two neurons and two output nodes with ReLU activation function. Here *x* is the input vector, $f_{ijc}(x)=w_{ijc}x+b_{ijc}$ with $i=$ the neuron number 1-2, $j=$ the step 1-3, and ReLU activation function $= max(0,f_{ijc}(x)$

As we introduce more inputs and neurons, the complexity of the NN increases greatly. Consequently, the number of parameters to be trained increases and requires more computational power. We illustrate this increased complexity in Figure 5 showing a NN with two inputs, two neurons, and two outputs.

To visualize the usefulness of NN, an Excel implementation was created using the Data Analysis Solver tool to optimize NN weights and biases that minimize error between the real and predicted data. In this way our approach to the nonlinear optimization problem is simpler than the MS-Excel implementation in (Zaiontz 2024) that solves an SIR problem using backpropagation formulas to optimize weights and bias.

### Application to Disease Dynamics with Data

The Susceptible-Infectious-Recovered (SIR) model is a well-known mathematical model (Kermack and McKendrick 1927) used in epidemiology to understand the spread of infectious diseases. SIR divides the population into three compartments: Susceptible (*S*) who are vulnerable to infection; Infectious (*I*) who are currently infected and can transmit the disease; and Recovered (*R*) who have recovered from the disease and are immune. The model is governed by the following set of differential equations in 2 that describe the rates at which individuals move from being susceptible to infectious, and from infectious to recovered. These rates are influenced by factors such as the transmission rate of the disease and the recovery rate.

2 $$\begin{aligned} \begin{aligned} \dfrac{\textrm{d}S}{\textrm{d}t}&= -\beta S \frac{I}{N}\\ \dfrac{\textrm{d}I}{\textrm{d}t}&= \beta S \frac{I}{N} - \sigma I\\ \dfrac{\textrm{d}R}{\textrm{d}t}&= \sigma I \end{aligned} \end{aligned}$$

where $\beta$ and $\sigma$ are the transmission and recovery rate, respectively. On the other hand, *N* is the total population such that $S(t) + I(t) + R(t) = N$ for all *t* in the domain.

**Fig. 6:**
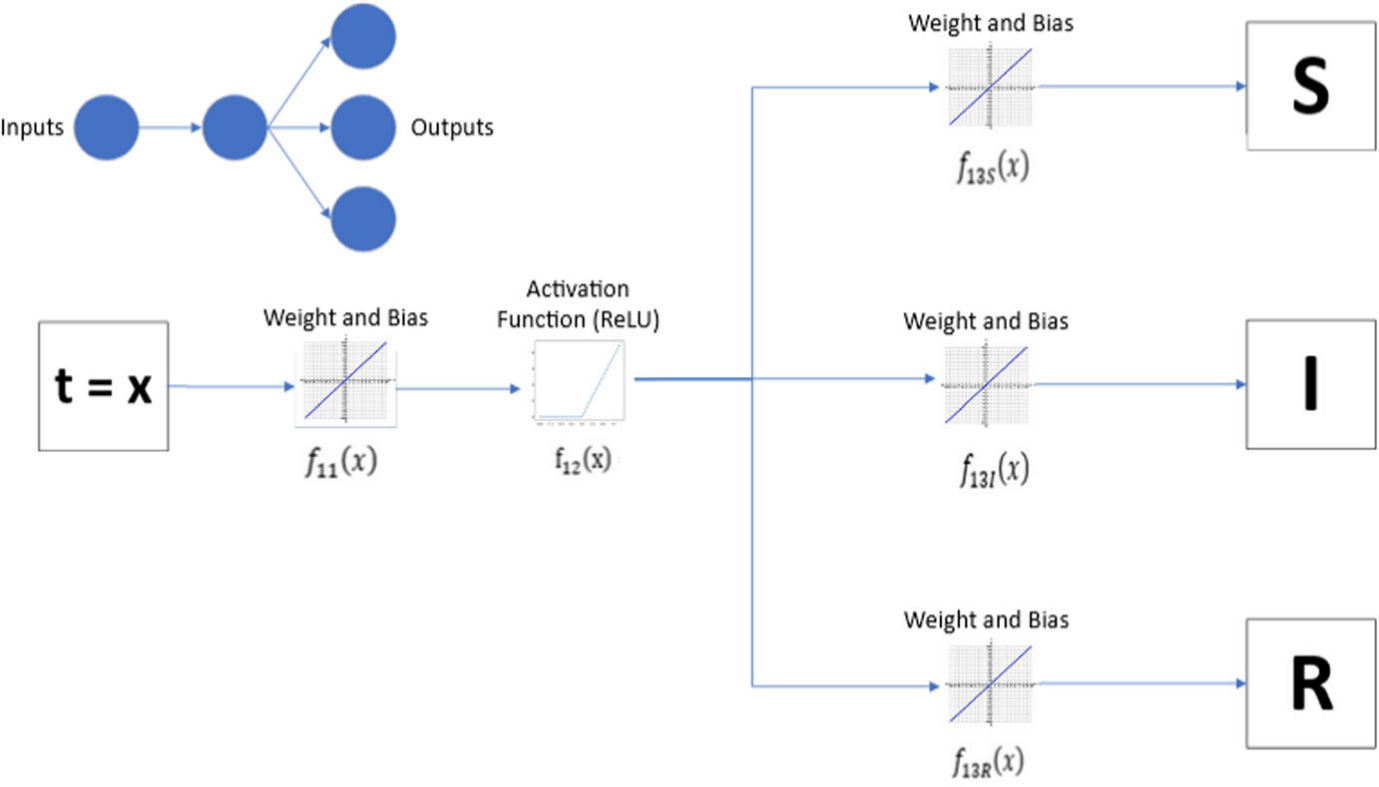
NN with one inputs node, one neuron and three output nodes with ReLU activation function. Here *x* is the input vector,$f_{ijc}(x)=w_{ijc}x+b_{ijc}$. Here $i=1$ is the neuron number, *j* is the step {1,2,3}, and ReLU activation function $=max(0,f_{ijc}(x))$

In the context of biomathematics, it is possible to apply NN in epidemiological models. The simplest model in Figure 6 shows an implementation of a NN with one neuron to solve the SIR model.

### Adding Physics to Neural Networks

Physics-informed neural networks differ from traditional neural networks by giving the network a system of governing equations (Raissi et al. 2019a), which in our case would be our models for data-driven learning. The network will obey the rules of this system of governing equations and help produce results that make sense in the terms of our models. (Raissi et al. 2019b) proposed a PINNs approach for model (2) where the differential residual of each equation is included in the loss function. Recently, (Shaier et al. 2022) proposed a complete framework to take advantage of the hidden physics of infectious diseases and infer the latent quantities of interest by approximating them using PINNs.

For model (2), let $u(t; \lambda ) = \left( S(t), I(t), R(t), \right) \in \mathbb {R}^3$ the function that we want to approximate through PINNs, and $\lambda = \left( \beta , \sigma \right) \in \mathbb {R}^2$ the vector of parameters related to the dynamics of the model. The training data has been discretized as $\{t^j, u^j\}_{j=0}^{N_{\text {data}}}$, where $N_{\text {data}}$ is the amount of training samples. The approximation is defined as $\widehat{u}(t; \lambda , \theta )$ where $\theta$ is the vector of learnable parameters defined by the architecture of the neural network used, for example, for a fully connected neural network $\theta$ corresponds to the concatenation of biases and weights. In practice, during the training process $\lambda$ are also considered learnable parameters, appending them to the vector of learnable parameters $\theta$. The loss function is a linear combination of the differential residuals (ode), initial conditions (ic) and training data residuals (data), see (3).

3 $$\begin{aligned} \mathcal {L}(\widehat{\lambda }, \widehat{\theta }) = \mathcal {L}_{\text {ode}}(\widehat{\lambda }, \widehat{\theta }) + \mathcal {L}_{\text {ic}}(\widehat{\lambda }, \widehat{\theta }) + \mathcal {L}_{\text {data}}(\widehat{\lambda }, \widehat{\theta }) \end{aligned}$$

where $\sigma _{\text {ode}}$, $\sigma _{\text {ic}}$ and $\sigma _{\text {data}}$ are hyper-parameters of the loss weights of the loss functions of the system of differential equations, initial conditions and training data, respectively. While the overall loss function is decomposed in other three parts, the loss function of differential residuals is expressed as:

$$\begin{aligned} \mathcal {L}_{\text {ode}}(\widehat{\lambda }, \widehat{\theta })&= \sigma _{\text {ode}, 1} \mathcal {L}_{S}(\widehat{\lambda }, \widehat{\theta }) + \sigma _{\text {ode}, 2} \mathcal {L}_{I}(\widehat{\lambda }, \widehat{\theta }) + \sigma _{\text {ode}, 3} \mathcal {L}_{R}(\widehat{\lambda }, \widehat{\theta }) \end{aligned}$$

where

4 $$\begin{aligned} \begin{aligned} \mathcal {L}_S&= \left( \dfrac{\textrm{d}S}{\textrm{d}t} + \beta S \frac{I}{N} \right) ^2 \\ \mathcal {L}_I&= \left( \dfrac{\textrm{d}I}{\textrm{d}t} -\beta S \frac{I}{N} + \sigma I \right) ^2 \\ \mathcal {L}_R&= \left( \dfrac{\textrm{d}R}{\textrm{d}t} - \sigma I \right) ^2 \end{aligned} \end{aligned}$$

The loss function corresponding to the data may be expressed as:

$$\begin{aligned} \mathcal {L}_{\text {data}}(\widehat{\lambda }, \widehat{\theta }) = \sum _{i=1}^3 \frac{\sigma _{\text {data}, i}}{N_{\text {data}}} \sum _{j=1}^{N_{\text {data}}} \left( u^j_i - \widehat{u}_i^j(\widehat{\lambda }, \widehat{\theta }) \right) ^2, \end{aligned}$$

and the loss function corresponding to the initial condition may be expressed as:

$$\begin{aligned} \mathcal {L}_{\text {ic}}(\widehat{\lambda }, \widehat{\theta }) = \sum _{i=1}^6 \sigma _{\text {ic}, i} \left( u^0_i - \widehat{u}_i^0(\widehat{\lambda }, \widehat{\theta }) \right) ^2 \end{aligned}$$

where $u_i$ for $i=1, \ldots , 3$ corresponds each component of $u(t ; \lambda )$, for example, $u_1$ corresponds to *S*, $u_2$ corresponds to *E* and $u_3$ that corresponds to *R*.

**Fig. 7:**
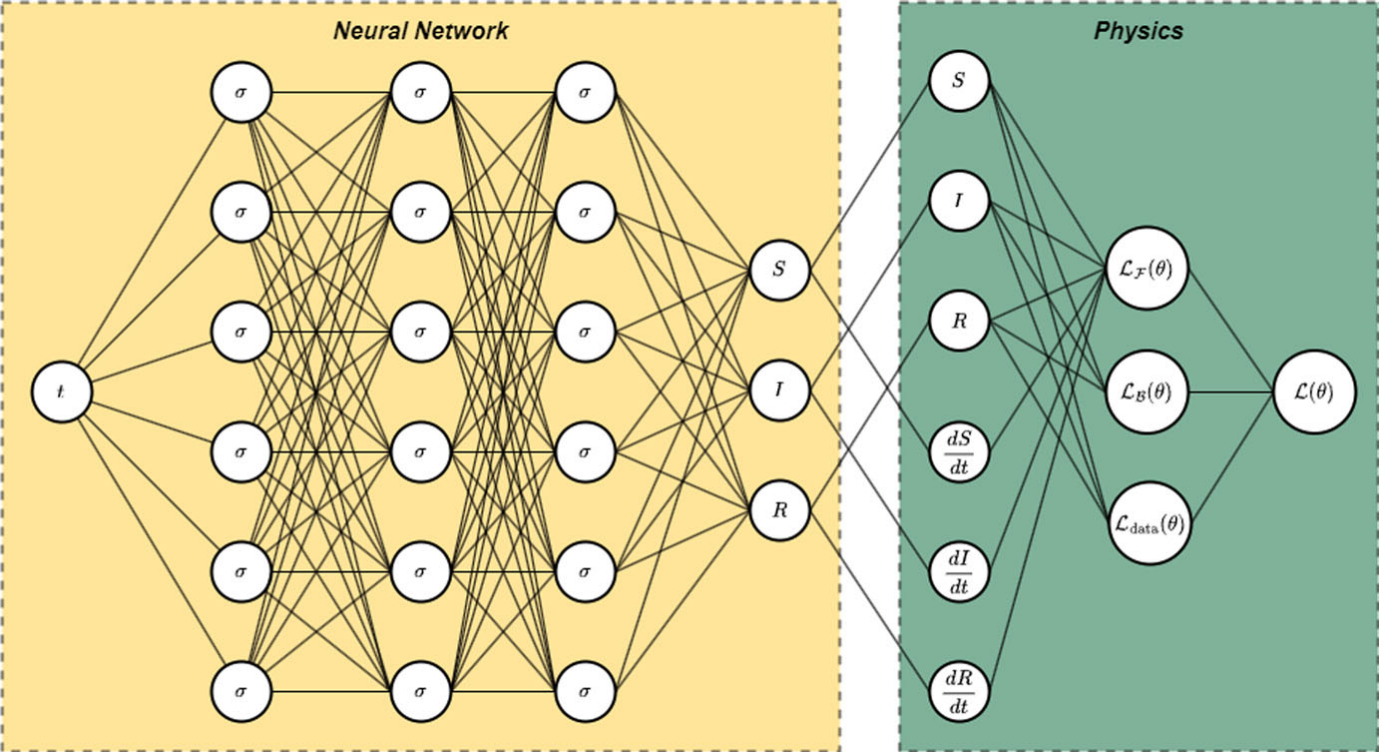
Physics-informed neural network architecture for model (2)

Figure 7 shows the architecture of the framework, where the input layer corresponds only to time. The output layer has three nodes, one per each compartment. Using automatic differentiation is possible to compute the derivatives of *S*, *I* and *R* numerically to feed the residual loss function. Algorithm 1 shows how to estimate $u(t; \lambda )$ and the parameters $\lambda$.

**Algorithm 1:**
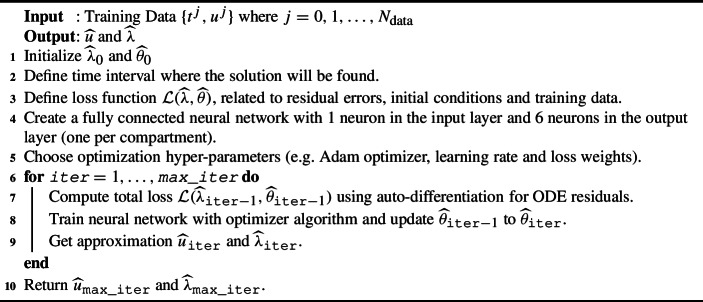
PINNs algorithm.

## Implementation of Neural Networks

### Simple Disease Informed Neural Network with MS-Excel

According to a survey in Statista, more than 1.3 million companies in United States uses MS-Excel (Excel) as part of their productivity tools (statista 2024) due to its availability, flexibility and user-friendly interface. The high demand for employees with Excel skills is evidenced in its inclusion in resume building tools such as Teal-HQ. Its availability through Office 365 make educational institutions incorporate Excel in their courses. Currently, neural networks are implemented in high-level programming languages such as Python, and Java Script, with a steep learning curve. Using the theory provided in this paper, it is possible to implement a simple-NN using Excel’s tools. In particular, the solver add-in implements a nonlinear optimization tool that helps users find the best possible solution for a nonlinear problem with multiple variables and constraints. Using the generalized reduced gradient (GRG) nonlinear solver with multi-start option we can minimize the classification error of a NN by finding optimal weight and bias parameters. The solver will minimize the mean squared error (MSE) between the NN predicted data and the real data in this supervised learning algorithm. The "solver" option is enabled in Excel’s settings and add-in section.

**Fig. 8:**
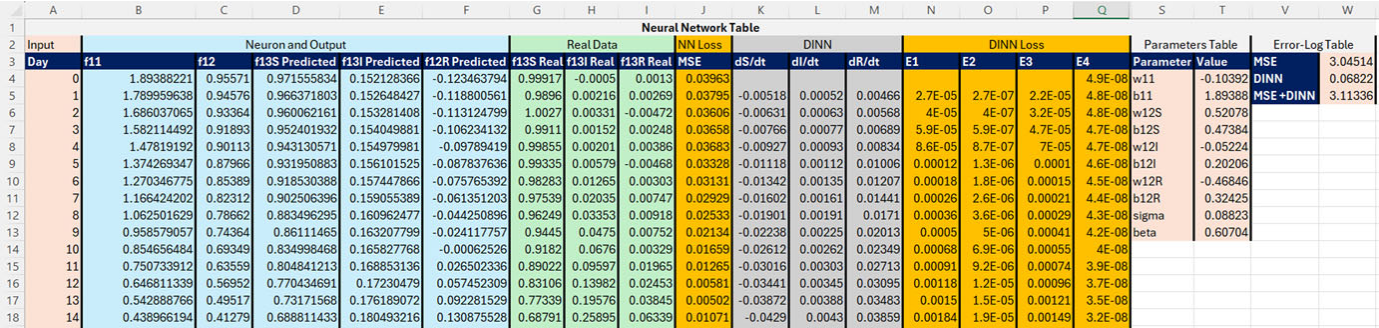
MS-Excel PINN table separating its components by bands of colored columns: Input (1 pink column), Neurons (5 blue columns), Data (3 green columns), NN loss function (1 orange column), DINN equations (3 gray columns), DINN loss function (4 orange columns), weights-bias parameters (2 pink columns) and error-log table (identified in dark blue rows: MSE, DINN and MSE + DINN

We will build a simple PINN applied to SIR Disease model denoted DINN using Figure 6 as an example. There are three tables used to explain the MS-Excel DINN implementation. In Figure 8 we illustrate the DINN table separating its components: “Input”/time in column A, “Neurons and Output” in columns B-F, “Real SIR Data” in columns G-I, “NN-Loss” computed as MSE of predictions in column J, “DINN” equations in columns K-M, “DINN Loss” functions in columns K-M as defined in (3), weights and bias “Parameters Table” in columns S-T and “error-log table” in V-W. Each Excel column represents a step of the algorithm and each row is the input value related to each day.

**Fig. 9:**
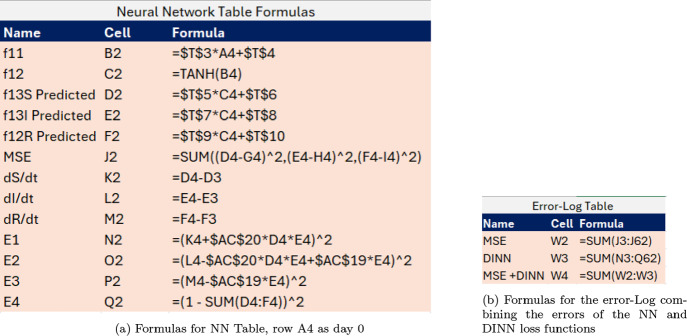
MS-Excel Formulas for the PINN Table

In Figure 9a we show the formulas included in the DINN Table using the same function notation illustrated in the one-node NN from Figure 6. These formulas use row 4 (A4) of the spreadsheet that corresponds to day 0. In Figure 9b we share the error formulas that combine both NN and DINN loss-function values. To calculate numerically the derivatives in cells K2, L2 and M2, we use finite-difference equations with a convenient time step of one. Once row 4 is implemented, the user can scroll down the formula to the remaining rows.

**Fig. 10:**
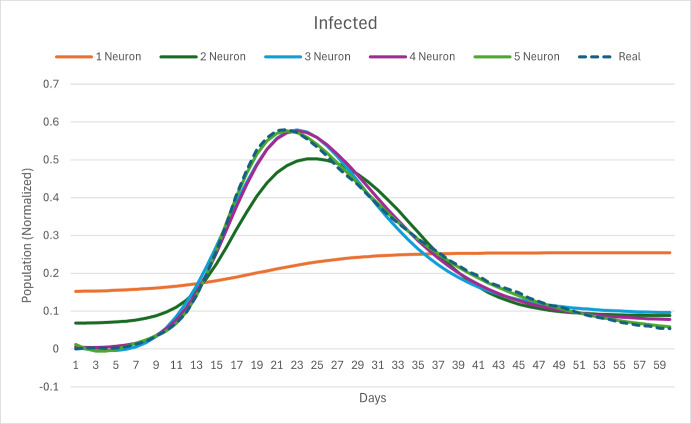
Excel results showing Infected curve for 1-5 neurons and dashed data values.

Using 60-days of normalized Susceptible (S), Infected (I) and Recovered (R) data, we optimized our one-neuron simple PINN. The calculated physics parameters of the equations are $\beta = 0.607$ and $\sigma = 0.088$, with a high relative error compared to the known parameters $\beta * = 0.5$ and $\sigma * \approx 0.0714$. If we increase the number of neurons from one to five, we can obtain the graphical results for the Infected variable in Figure 10.

**Fig. 11:**
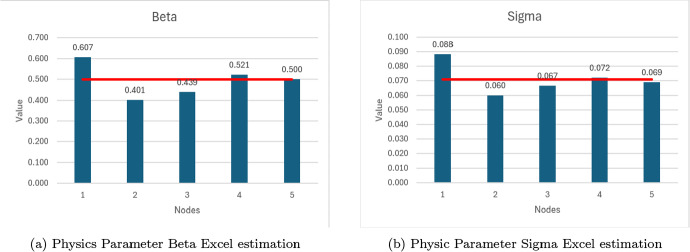
Parameter estimation in MS-Excel, with 1-5 nodes, (a) Beta = 0.5, (b) Sigma = 0.0714

We compare the physics parameter estimation of $\beta$ and $\sigma$ in Figures 11a and 11b.

**Fig. 12:**
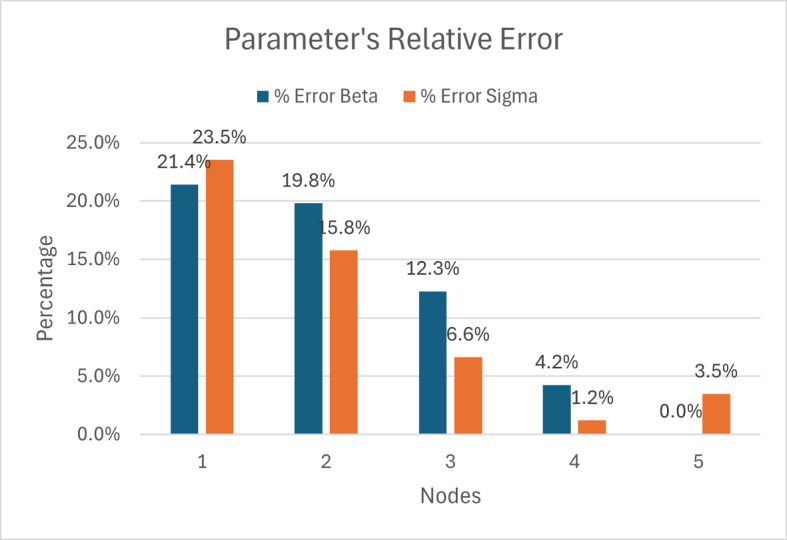
Parameter Relative Error

The relative error of both physics parameters for increasing NN nodes is summarized in Figure 12. We observe that there is an inverse relation between the relative error and the number of neurons. With a sufficient amount of data, we can increase the number of neurons until we get less than 0.05 relative error in our parameters.

Graphs for Susceptible and Recovered variables are shared as Supplementary Material B in Ogueda-Oliva et al. (2025). Also a step by step Excel implementation of the "Simple-PINN" is explained and we share the Excel files for further exploration.

Although we have obtained a successful Excel implementation, this is due in part to the small dimensions of our problem. We used a simulation with 60 days, very small quantity of data and up to five nodes. In larger problems with large amount of data and large number of nodes and parameters for optimization, Excel will be less efficient or will exceed its capacity. When a better scalable model is needed, we use the Python implementation discussed next.

### Disease Informed Neural Networks with Python

In this sub-section we discuss how to implement Algorithm 1 on the system of equations (2). Data has been generated using a Runge-Kutta implementation with values of $\beta = 0.5$, $\sigma =1/14$, $N=1000$ and with initial condition $S(0)=999$, $I(0)=1$ and $R(0)=0$. Afterward, a $1\%$ white noise was added to each compartment. For the following numerical experiments, we use Python as our main programming language, relying heavily on the DeepXDE package (Lu et al. 2021) with PyTorch as the automatic differentiation backend. The implementation architecture is a fully connected neural network with 3 hidden layers of 64 neurons and ReLU as activation function. The optimizer used was Adams with a learning rate of 0.001 and we did 30,000 full-batch iterations. The source code is available online in Ogueda-Oliva et al. (2025).

#### Python Implementation

Start by importing the packages: 
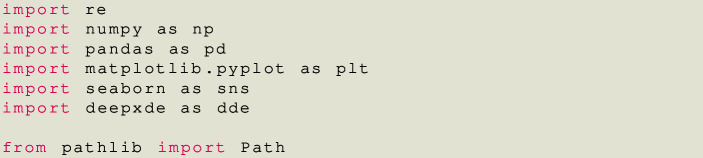


Now, declare *N* as the total population (1000) and the real values of $\beta$, $\sigma$ to use them at the end for error calculations. 
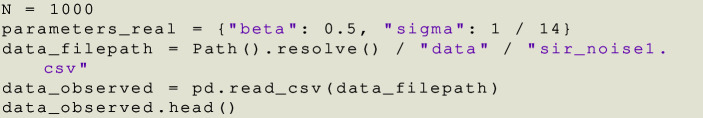

**Table 2:** First five rows of the synthetic data with noise applied

t	S	I	R
0	999.17	-0.52	1.30
1	989.60	2.16	2.69
2	1002.70	3.31	-4.72
3	991.10	1.52	2.48
4	998.55	2.01	3.86

Extract the observed values of time and each compartment as column vectors for using them as training data. 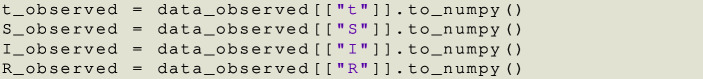


Initialize $$\beta $$ and $$\sigma $$: 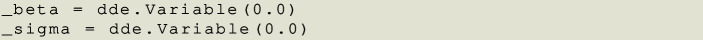


Now we can define the differential residuals, a function that in our case takes as input t (time) and an array y that contains each compartment, for a total of four columns. This function returns the residuals of each compartment. 
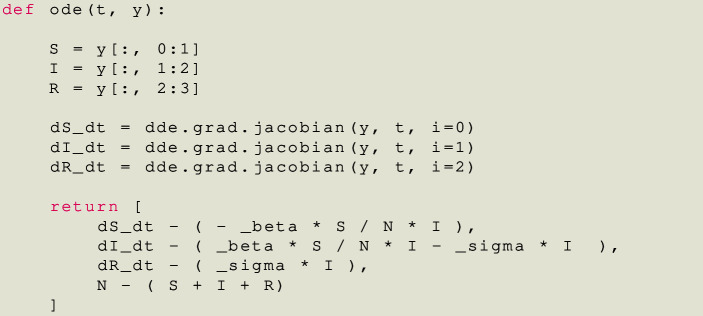


Notice how each derivative correspond to the columns of its compartment. For example, the first column of the array y corresponds to the Susceptible (*S*(*t*)) compartment and the derivative $\dot{S}(t)=\frac{\text {d}S}{\text {d}t}$ is declared with $i=0$, referencing the first component.

It is also important to define the domain of the differential equation that in this case is only a time-domain. 
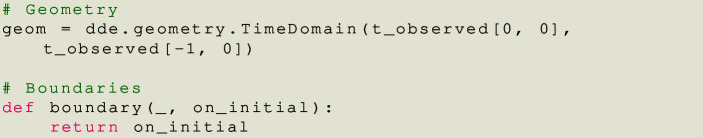


The observed data of each compartment has to coincide with the component of its derivative. For example, notice that in the following block of code the component of observed_S is 0. 
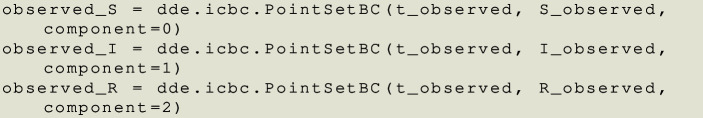


We have the main elements of the differential equations. Here it is important to put them together in one single object. 
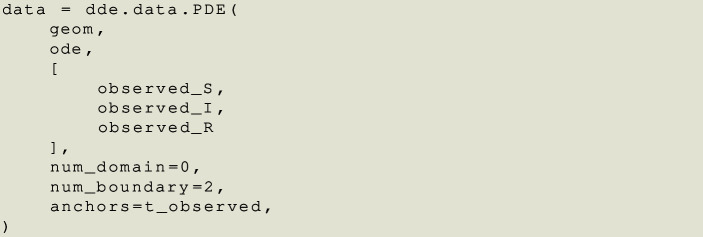


The previous code takes a domain (geom), its differential residuals (ode) and the observed data. The argument num_domain=0 means that in the training process we are not sampling any element inside the domain, except for the values of time that have observed data (anchors=t_observed).

Now we have to define the network architecture, which is basically one line of code and our highest complexity run. 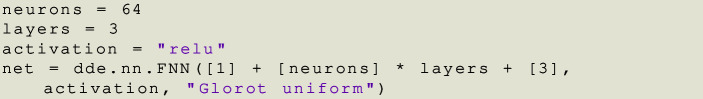


Since we are solving an inverse problem, we declare that $\beta$ and $\sigma$ are also learnable parameters. More importantly, we want to keep track of the learning process of both parameters. 
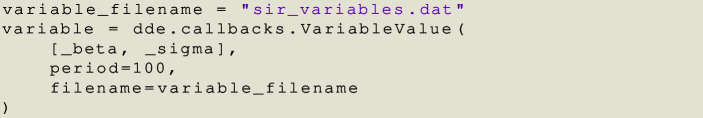


Finally, we compile and train the model. 
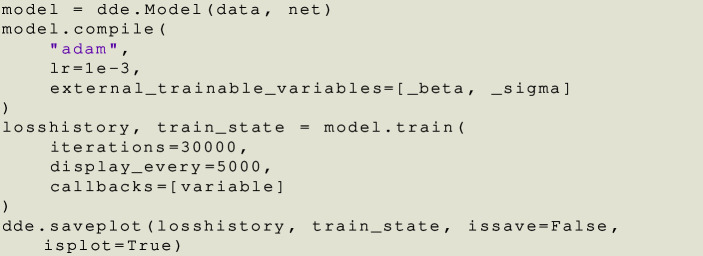

**Fig. 13:**
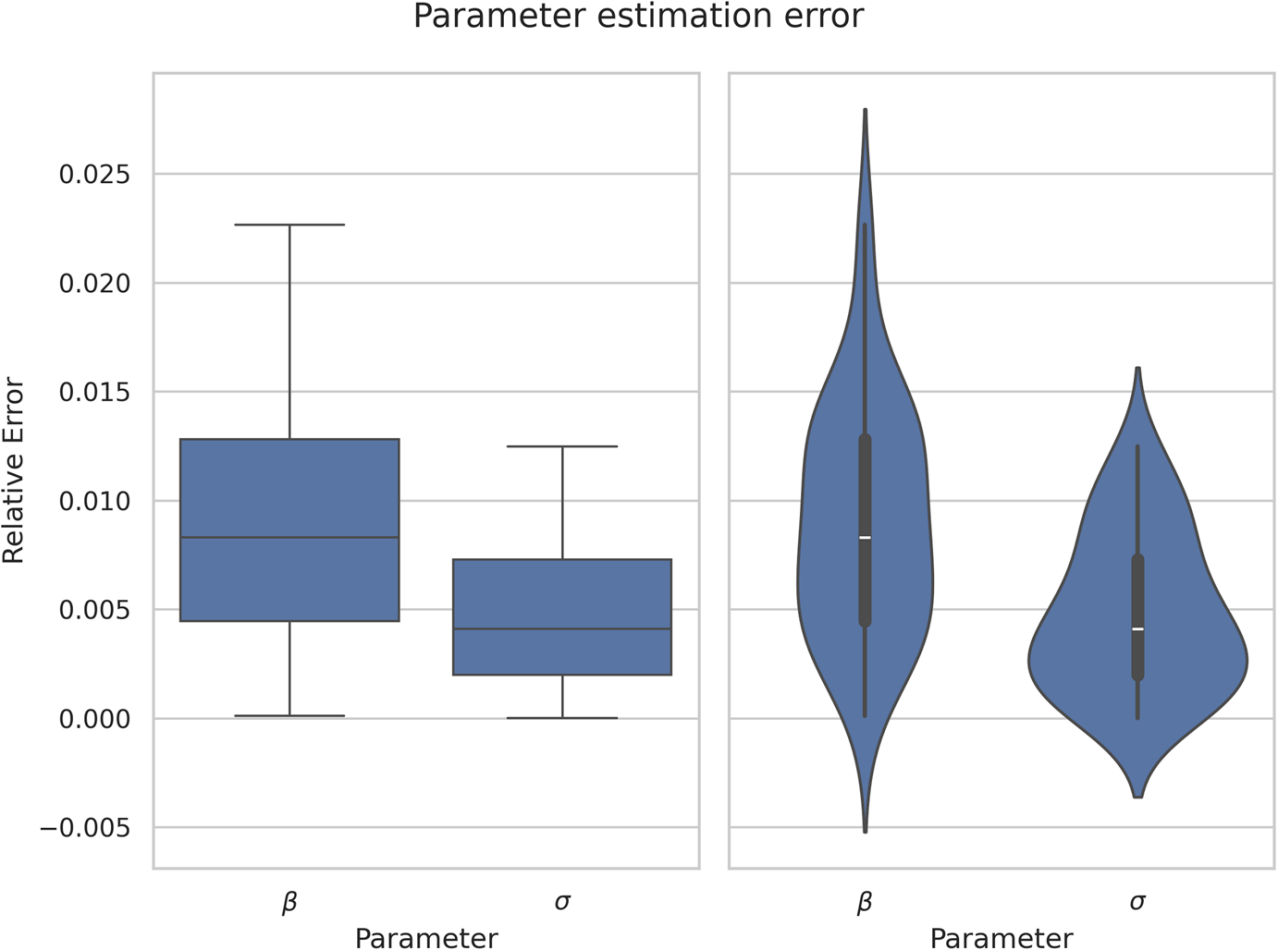
(Left) Boxplot of relative errors of $\beta$ and $\sigma$ with 30 executions of PINNs. (Right) Same but Violinplot showing the median as a white dot, the quartile range as a bar and the distribution of the relative error flipped and mirrored with respect to the bar.

#### Robustness

Neural networks can potentially be very susceptible to initialization (Glorot and Bengio 2010). Hence, we fixed the initial guesses of $\beta$ and $\sigma$ as zero and set the NN weights initialization of the fully connected neural network with Glorot Uniform initialization (also known as Xavier Uniform). We ran the algorithm 30 times to study its robustness with different weight initialization. Figure 13 shows the spread of the relative error of parameters $\beta$ and $\sigma$ for 30 different executions of PINNs. Notice that even when the data has a $1\%$ error included, in general the framework is able to obtain very consistent and small relative errors for both parameters. The average prediction for these 30 runs was $\widehat{\beta } = 0.4971 \approx 0.5$ and $\widehat{\sigma } = 0.0713 \approx 0.0714$, with a standard deviation of 0.004446 and 0.000420, respectively.

**Fig. 14:**
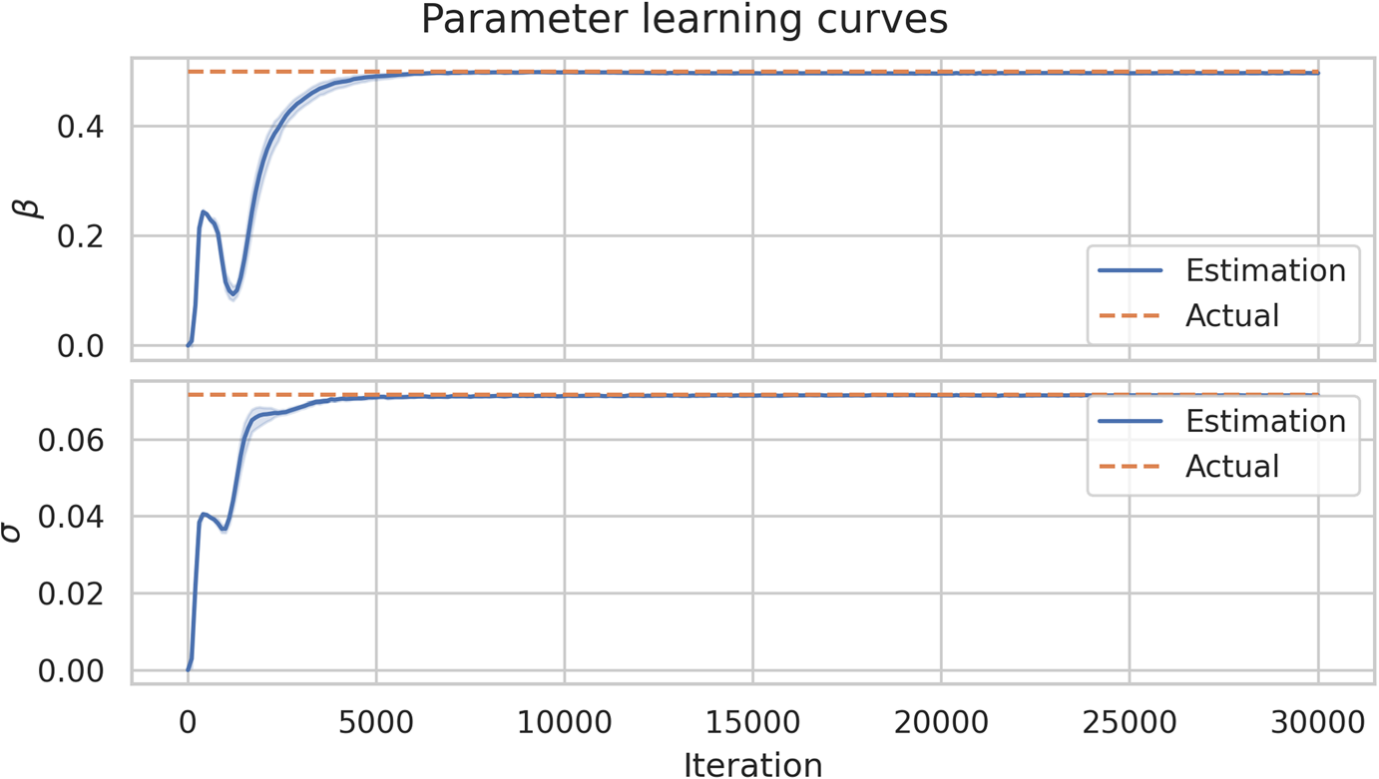
Learning curves for $\beta$ and $\sigma$ with a $95\%$ confidence interval band with 30 executions of PINNs. Blue lines correspond to the average estimated value while the orange lines are the actual value of each parameter

Figure 14 shows the evolution of the estimated values of the parameters $\beta$ and $\sigma$ by iterations. We can notice how at the beginning of the curve the values oscillated, this is mostly because the first iterations the networks have not fit good weights and biases, so even the predictions of the compartments are not accurate. However, we can observe how both learning curves take a increasing behavior after a few hundred iterations. Finally, it converges quickly to the actual value around 5000 iterations. Each of these 30 experiments took minutes on a personal computer with a single GPU in order to accelerate the backward propagation step of the optimizer. Apriori the user does not know how many iterations would be enough to achieve a desired accuracy; however, it is important to note that in deep learning applications the number of iterations could be millions. PINNs take advantage of the physics which allows training to be performed with a few thousand iterations.

## Discussion and Conclusion

In this work, a C-MATH with NN educational framework has been discussed and illustrated including a graphical introduction to NN with SIR epidemic modeling equations (PINN). The style is mostly pedagogical in the spirit of providing tools for our educators that are transforming education through AI and mathematical biology integration. Using Literate Programming instruction we share the MS-Excel and Python programs files to solve the SIR equations and estimate the infection and recovery parameters, $\beta$ and $\sigma$ respectively known from a COVID-19 synthetic data. The scalability limitation in Excel to program a large number of nodes and layers restricted the implementation to a simple-PINN with one hidden layer architecture. The simple architecture is suitable for many smaller programs that are of interest for example to community partners and small businesses that use also MS-Excel. Results for a one hidden layer with 5 neurons feed-forward PINN show that nonlinear optimization solvers that minimize loss functions and error can do a very good job in this simple architecture. Weights and bias parameters, and physics$\sigma , \beta$ parameters were obtained with a small error defining the network as a non-linear optimization problem. Excel’s non-linear solver, generalized reduced gradient (GRG2) optimization has compared favorably over gradient descent back propagation in the past in (Hung and Denton 1993) with multiple initializations to improve the loss function minimization. Nonetheless, the Python multi-perceptron NN scales very easily to 3 layers and 64 neurons. This is not recommended for Excel implementation because it will greatly multiply the number formulas defeating its simplicity purpose. The computational power of our times paired with the efficient back propagation algorithm make Python and R implementation attractive for general learning features and relationships that does not require previous initialization knowledge and reduce output classification/prediction errors at the cost of a more complex architecture.

The motivation of the work and tools shared is educational. We propose that low code platforms as MS-Excel are great for understanding the implementation behind complex mechanisms, in particular, artificial neural networks. Students usually are familiarized with spreadsheets software, and otherwise they are able to understand the required functionalities on the fly. This enables educators to teach basic biomathematics applications and theory with engaging technology minimizing the learning-curve inherent to coding. It is also important to point out that low code implementation is an alternative to many real-world applications that have small amount of data available. However, for those students with coding background or interests in popular languages such as Python, the shared files with the AI assistants are good options to learn and explore this powerful and scalable tools that adapt easily to larger problems and datasets. In this work we show an implementation of Python on top of different scientific packages. This approach has a better efficiency in scalability in two ways, development time and execution time. The popular neural network framework on the Python ecosystem is flexible enough to change the number of nodes and layers in a few lines, unlike the low code MS-Excel alternative. Another advantage of modern computers is that the NN training process can be parallelized taking advantage of GPU hardware, which can reduce the execution times by several orders of magnitude (Raina et al. 2009), making it possible to train neural networks that could not be feasible in realistic time ranges using CPUs.

There is a potential danger in educating by using technology blindly and also in disregarding the advances of technology in Biomathematics education. Mathematical Biology provides a context that is meaningful for the learner in their sociobiological environment supporting intrinsic motivation to learn the mathematics of technology to uncover the mechanisms of biological systems. The C-MATH-NN framework uses Literate Programming pedagogy integrated in a book that seamlessly champions meaningful learning of AI-, computational literacy, and Biomathematics for our next-generation. of modules that seamlessly champion AI, computational literacy, and biomathematics context for our next-generation to contribute knowledge for social good.

The C-MATH-NN framework presented in this work demonstrates how the integration of Technological, Pedagogical, and Content Knowledge (TPACK) can be both balanced and purposeful in designing learning experiences for the age of AI. Rather than treating technology as an external tool or optional enhancement, C-MATH-NN positions it as an essential, embedded component of the learning process. This approach reflects the core principles of the TPACK framework while pushing its application into new, interdisciplinary, and future-oriented directions. By combining accessible technologies like Excel and Python with pedagogical strategies rooted in Literate Programming, and by engaging learners in complex, real-world content such as epidemiological modeling and neural networks, C-MATH-NN serves as a scalable model for AI-era education. It illustrates how educators can move beyond superficial uses of technology to create transformative, interactive, and skill-oriented learning environments. As such, C-MATH-NN not only aligns with TPACK but also expands its practical relevance, offering a path toward equipping students with the knowledge and competencies required in an increasingly AI-driven world.

In order to support educators we share the developed resources, MS-Excel files and Python code, to facilitate the acquisition of this framework and its technology for all to explore in Ogueda-Oliva et al. (2025).

## Data Availability

This work uses synthetic data to train the NN with physics equations and recover the physics parameters of the differential equations modeling a disease dynamics problem. The objective of using synthetic data from known equations is to quantify the parameter estimation error as the difference between the NN approximation and the known equations parameters used to generate the synthetic data. The first five rows of the generated synthetic data with 1 % noise are summarized in 2. This data set has been used to obtain results from the Excel and Python implementation of the PINN presented in figures 8, 10, 11, 12, 13 and 14. The data set is available on the project’s GitHub repository in Ogueda-Oliva (2025).

## Footnotes

1 https://aoguedao.github.io/teaching-ml-diffeq/
